# “Establishing the criterion validity of the interRAI Check-Up Self-Report instrument”

**DOI:** 10.1186/s12877-020-01659-9

**Published:** 2020-07-29

**Authors:** Leon N. Geffen, Gabrielle Kelly, John N. Morris, Sophie Hogeveen, John Hirdes

**Affiliations:** 1 Samson Institute for Ageing Research, University of Cape Town, Cape Town, South Africa; 2 Samson Institute for Ageing Research, 234 Upper Buitenkant Street, Cape Town, 8001 South Africa; 3 Marcus Institute for Aging Research, Boston, MA USA; 4 Women’s College Hospital Institute for Health System Solutions and Virtual Care; McMaster Institute for Research on Aging, Hamilton, Canada; 5 School of Public Health and Health Systems, University of Waterloo, Waterloo, Canada

**Keywords:** interRAI, Validity, Geriatric assessment, Comprehensive assessment, South Africa

## Abstract

**Background:**

Low and middle-income countries have growing older populations and could benefit from the use of multi-domain geriatric assessments in overcoming the challenge of providing quality health services to older persons. This paper reports on the outcomes of a study carried out in Cape Town, South Africa on the validity of the *interRAI Check-Up Self-Report* instrument, a multi-domain assessment instrument designed to screen older persons in primary health settings. This is the first criterion validity study of the instrument. The instrument is designed to identify specific health problems and needs, including psychosocial or cognition problems and issues related to functional decline. The interRAI Check-Up Self-Report is designed to be compatible with the clinician administered instruments in the interRAI suite of assessments, but the validity of the instrument against clinician ratings has not yet been established. We therefore sought to establish whether community health workers, rather than trained healthcare professionals could reliably administer the self-report instrument to older persons.

**Methods:**

We evaluated the criterion validity of the self-report instrument through comparison to assessments completed by a clinician assessor. A total of 112 participants, aged 60 or older were recruited from 7 seniors clubs in Khayelitsha, Cape Town. Each participant was assessed by one of two previously untrained, non-healthcare personnel using the Check-Up Self-report version and again by a trained assessor using the clinician version of the interRAI Check-Up within 48 h. Our analyses focused on the degree of agreement between the self-reported and clinician-rated versions of the Check-Up based on the simple or weighted kappa values for the two types of ratings. Binary variables used simple kappas, and ordinal variables with three or more levels were examined using weighted kappas with Fleiss-Cohen weights.

**Results:**

Based on Cohen’s Kappa values, we were able to establish that high levels of agreement existed between clinical assessors and lay interviewers, indicating that the instrument can be validly administered by community health workers without formal healthcare training. 13% of items had kappa values ranging between 0.10 and 0.39; 51% of items had kappa values between 0.4 and 0.69; and 36% of items had values of between 0.70 and 1.00.

**Conclusion:**

Our findings indicate that there is potential for the Check-Up Self-Report instrument to be implemented in under-resourced health systems such as South Africa’s.

## Background

Simple low-cost solutions are needed to provide quality health and social care to the rapidly growing number of older persons in Low and Middle Income countries (LMICs) [1]. The World Health Organisation has identified the optimisation of intrinsic capacity and functional ability through early intervention at the community or primary care level as key to healthy ageing, which will reduce healthcare costs and care dependency [2]. Intrinsic capacity is defined by the WHO as “the composite of all the physical and mental capacities that an individual can draw on,” while functional ability consists of the intrinsic capacity of the individual, the environment of the individual and the interactions between them.

Multi-domain geriatric assessment instruments have been shown to be effective in understanding function and identifying deterioration in intrinsic and functional ability, making medical diagnoses, identifying cognition or psychosocial problems and facilitating access to appropriate medical care and social support [3–8]. Identifying individual needs allows for the provision of appropriate early and multi-disciplinary interventions that have the potential to reverse or slow losses in intrinsic capacity and prevent associated declines in functional ability, improve health outcomes and well-being and potentially reduce the individual and societal effects of frailty, disability and dependence [9–11].

However, validated multi-domain geriatric instruments typically require specialised expertise to perform [12]. Therefore, these may not be practical to use in primary care settings, particularly in LMICs that lack the health system resources required to carry out clinician-administered assessments on large populations. In an under-resourced health system, assessment instruments that can be used in the community by non-healthcare workers to screen patients may be more feasible to use at scale.

In this study, we test the validity of the *interRAI Check-Up Self-Report*, a geriatric assessment instrument designed to be used by non-healthcare professionals or by the patient themselves (provided they have high enough levels of literacy and a minimum level of cognitive function) to identify losses in intrinsic capacity at the primary care level. Losses of intrinsic capacity are measured in terms of both physical and mental capacities and are determined in relation to the ability to complete certain tasks (e.g. climb stairs) or the presence of particular symptoms in a pre-defined period. Hearing and vision loss, difficulties with communication, mood, cognition, ability to carry out instrumental activities of daily living, as well as fatigue, falls, breathlessness and continence are all considered.

We compared the self-report ratings on this instrument with subsequent clinician ratings as the gold standard. This instrument is the first self-report based assessment in the interRAI family of instruments, an integrated suite of comprehensive multi-dimensional assessments and screeners for use with a number of vulnerable populations (including older persons), developed by a not-for-profit network of health researchers from over 30 countries [13–16].

All adult instruments are built on a minimum set common set of common items, as well as specialised items relevant to particular contexts and patient groups, and are designed to track persons longitudinally over time and across multiple care settings. These instruments have been continuously improved and validated against other commonly used instruments for use in long-term care, acute and post-acute care, home care, mental health, palliative and community settings [16–25]. The item domains of these instruments have been shown to have good inter-rater reliability [16] and have been adopted internationally, including countries in North America, Europe, the Middle East, Australasia and East and South East Asia [26, 27].

Third-generation geriatric assessment instruments such as those in the interRAI suite have several advantages over first and second-generation instruments. First-generation instruments are typically single-domain instruments that need to be conducted separately. Second generation instruments such as the Minimum Geriatric Screening Tool (MGST) include all geriatric domains, are setting-specific and have been validated in each specific setting [13, 28]. However, in using these instruments the design parameters require that healthcare professionals use informed, clinical judgement to decide on which components of the instrument to select [28]. Third-generation instruments such as the interRAI suite of assessments use a more focused, standardised set of clinical items (or minimum dataset) and scales attached to the various domains to allow for data transfer across multiple settings which allow patients to be tracked longitudinally and in different settings [15]. If patient registers as high-risk for a certain item (e.g. falls), Clinical Assessment Protocols are triggered, which provide guidelines for further examination and treatment. These protocols provide information on evidence-based approaches to geriatric care to inform the interpretation and response to the assessment results [13, 28, 29].

The interRAI Check-Up instrument is based on a sub-set of around 90 items from the interRAI Home Care instrument [15, 16, 30]. The interRAI home care (interRAI-HC) is probably the most well-researched and supported community-based multi-domain assessment globally [6]. Two recent systematic reviews of the interRAI Home Care instrument have shown that it can be supportive tool for quality care planning to identify problems and risk situations and can foster collaboration between healthcare professionals within and across care settings and improve communication between caregivers [17, 24]. Together with case management, it has been shown to reduce hospital admissions, length of stay, and thus, reduce additional expenditure and associated costs [5, 17, 24].

The Check-Up instrument was specifically designed to address the needs and status of older persons living in the community including those receiving primary care services to inform individual care planning. The aim of this instrument is to identify the need for further assessment, medical intervention, care or psychosocial support. The Check-Up is relatively quick to administer and therefore acts as a bedrock instrument in settings where long, detailed assessments are unnecessary or not feasible. The instrument is designed for repeated use and declines in capacity can be picked up through multiple assessments over time.

In high-income countries, the clinician administered version of the Check-Up instrument is typically done by a trained nurse or social worker using all sources of information, including direct interviews and observations of the person being assessed, interviews with formal and informal caregivers, and a review of available clinical charts. The assessor considers all available information and then exercises clinical judgment about what would be the most appropriate response for a given item. However, health care services in LMICs and other resource constrained communities may lack the professional resources that are required to conduct multi-domain assessments on a large scale basis. However, the recently-developed self-report version of the Check-Up has the potential to be used in LMICs, where low-skilled but literate community health workers can administer the instrument in primary care settings (or it can be self-administered). Nursing and medical staff can be provided with the output summaries, giving them the information and guidance needed to provide better care to the older persons they treat. The results of the self-report tool can be used to flag the subset of individuals most in need of a comprehensive assessment by health professionals. The instrument can also be used to gather much-needed population-level data on community-dwelling older persons in LMICs so as to inform policymakers and planners.

The self-report version of the Check-Up uses fixed narrative questions and responses that convert the corresponding clinician-rated items from the longer interRAI Home Care into survey style questions that can be self-administered or asked by a lay interviewer. These items retain the time frames, exclusion/inclusion criteria, item definitions and examples from the interRAI Home Care, but ask questions in a format accessible to lay persons. As a result, responses to the self-report instrument can be used to derive many of the scales and care planning algorithms found in the clinician-administered instruments in the interRAI suite.

The validity of the Check-Up self-report instrument against clinician ratings has not yet been established. We therefore sought to establish the capacity of lay assessors without any health expertise to accurately administer the self-report instrument to older persons. This formed part of a larger study using the Check-Up self-report in four communities in Cape Town, South Africa.

## Methods

We evaluated the criterion validity of the Check-Up self-report instrument by examining the level of agreement between self-report assessments completed to lay interviewers and the clinician version of the assessment using all sources of information completed by a clinician assessor. Cohen’s Kappa values were used to quantify the level of agreement between the two approaches.

### Participants and setting

A total of 112 participants, aged 60 or older were recruited from 7 seniors clubs in Khayelitsha, a peri-urban, low-income area of Cape Town. Khayelitsha is a high density area with a total population of 391,749, approximately 30 km from Cape Town [31]. The population is predominantly disadvantaged black, isiXhosa speaking South Africans with limited resources and limited access to formal healthcare services. The population is relatively youthful, with people aged 65+ comprising only 1.6% of the population (6268 people) according to 2011 census data [31].^1^ Unemployment in the area is extremely high and exceeds 38%. A quarter of the households have incomes of less than USD300. Living standards are low - over 35% of dwellings are informal shelters and many people have no piped water to their homes (65%), no flush toilets (28%), or electricity for lighting (19%) [31]. Persons over the age of 60 in South Africa have access to free primary health care in the public sector, including free medication. However, services are not geared to meet the needs of older persons. Nurse practitioners and doctors who provide care in the crowded clinics in low income areas do not have the time to attend to the complex needs of older persons [33].

All participants were members of seniors’ clubs, which act as a safe space for older persons to socialise in community settings on a daily or otherwise regular basis. Many of these clubs are run by non-profit organisations and are funded by the Department of Social Development. Clubs generally offer meals, exercise activities, access to social work and health promotion services, and present opportunities for participation in income generation projects. Some clubs also offer home visit services to participants too frail or unwell to attend the clubs. Three of the clubs in the study were formal clubs run by non-profits, while the others were informal community initiatives in the process of seeking government funding. These clubs generally vary in size from 10 to 50 members and interest in participation is generated via word of mouth.

### Instrument

For the South African pilot, the self-report Check-Up was translated from English into isiXhosa and Afrikaans, the key languages spoken in the study area. The instruments were translated by first-language speakers and then reverse translated by independent third parties who were also fluent in the language. If there was any conflict, the two translators met to resolve the issue. The instruments were also given to fieldworkers fluent in the vernacular to ensure that the meaning and context of the question was preserved. Fieldworkers also provided further feedback after using the instrument in the field and, after discussion with the translation team, the translations of the instrument were updated as necessary.

### Validation methodology

Participants were identified through a convenience sample, based on their interest in participating in the study after a visit from a fieldworker who explained the study to each club. The sample size (112) is sufficiently large to establish criterion validity using Cohen’s Kappa, which establishes the level of agreement between lay interviewers and clinician assessors taking into account the possibility of the agreement occurring by chance.

The trained assessors were responsible for obtaining informed written consent from all participants. All participants were assessed at club facilities, except for 24 participants who were no longer able to participate in club activities and were assessed at home. Each participant was provided with a small shopping voucher to thank them for their time; however, to avoid pressuring people with low incomes to participate, this was not advertised to participants until they had completed both assessments. Once patients consented, the Check-Up self-report instrument was administered by a lay interviewer employed for the purposes of the study and within 48 h, the clinician version of the instrument was administered by a clinical assessor. The “lay interviewers” had no healthcare background, were given 8 h of training each and were instructed to record the response as the patient reported them and not by their own observation. The “clinician assessor” is a health systems researcher working in the area of ageing and received 40 h of training on the clinical instrument and used her own judgement to make observations based on all sources of information available. For example, if someone reported there was no pain, but the assessor picked up that there was pain based on patient behaviour and further engagement, she might have recorded a different response to the research participant. On the other hand, the lay interviewers recorded only the person’s responses without making their own inferences about what answers would be correct.

The second assessment was completed within 48 h of the first assessment to minimise the chance that the condition of the participant would change between assessments. Assessors were prohibited from discussing the case with the interviewers, did not to exchange information and were blinded to the results of others.

Although assessment times were not measured with the accuracy we had hoped, when interviewers were familiar with the instrument, assessment times were about 30 min. This corresponds directly with a Canadian study of the interRAI Check Up Self Report which found that completion times were just below half an hour when self-administered or when a lay interviewer was used [34].

Our analyses focused on the degree of agreement between the self-reported and clinician-rated versions of the Check-Up based on the simple or weighted kappa values for the two types of ratings. Binary variables used simple kappas, and ordinal variables with three or more levels were examined using weighted kappas with Fleiss-Cohen weights. In cases where the observed response distributions did not match, responses were collapsed for the self-report or clinician-rated items in order to allow kappas to be calculated. In the case of extremely skewed distributions, response levels were collapsed in order to provide more stable kappa estimates. However, some binary items with highly skewed distributions (e.g., less than 5 % in a response level) could not be collapsed, which may result in unstable kappa estimates. Items with no variance (i.e., all observations with a single value) were excluded from the analyses.

### Ethics approval

Ethics approval for the study was obtained from the University of Cape Town’s Health Research Ethics Committee (HREC Ref: 790/2017).

## Results

### Description of sample

As Table 1 below shows, of the sample of 112 participants, 84% of participants were female and the average age of participants was 70.1 years. The majority (51%) of participants were widowed, while only 24% of participants were married or had a partner at the time of the study. None of the participants lived alone or exclusively with their spouse. The majority of participants (57%) reported living with relatives other than their immediate family or spouse. 63% of participants reported making trade-offs among purchasing adequate food, shelter, clothing, prescribed medications, sufficient home heat or cooling, or necessary health care or home care within the last 30 days because of limited funds. The high rate of trade-offs reported is consistent with the high level of poverty in the Khayelitsha area and the tendency of older persons to use their state pensions to support unemployed family members and grandchildren. Few participants reported themselves to be in excellent or good health, with the majority (71%) reporting that they were in fair to poor health.

**Table 1:** Sample description^a^

**Average Age**	70.1
**Gender**	
Female	94 (84%)
Male	18 (16%)
**Ethnicity**	
Black African	112 (100%)
**Trade-offs**	
No	41 (37%)
Yes	71 (63%)
**Marital status**	
Never married	19 (17%)
Married	27 (24%)
Partner	0 (0%)
Widowed	57 (51%)
Separated	5 (4.5%)
Divorced	4 (3.5%)
**Living arrangements**	
With spouse/ partner & others	19 (17%)
With child – not spouse/partner	20 (18%)
With siblings	3 (3%)
With other relatives.	64 (57%)
With non-relatives.	1 (1%)
Not answered.	5 (4%)
**Self-rated health**	
Excellent	3 (3%)
Good	29 (26%)
Fair	61 (54%)
Poor	19 (17%)

^a^Based on clinician assessment

Table 2 shows the average simple and weighted kappas for the items in the self-report Check-Up, as well as the number of items in the domain used to calculate the average. The literature on inter-rater reliability uses a conventional cut-off of 0.40 for acceptable inter-rater reliability; however, it should be noted that these analyses speak more to the clinical validity of items than reliability. A comparison between two lay interviewers would have provided evidence about inter-rater reliability, but the comparison of lay interviewer findings with those of clinicians pertains to criterion validity. Based on the 0.40 cut-off, seven of the nine domain areas had average kappa values that were at least acceptable. Three domains had excellent average kappa values above 0.65 and two fell below the 0.40 cut-off.

**Table 2:** Average Kappa or Weighted Kappa values for interRAI Self-Reported Check-Up items compared with clinician administered ratings

Section	Number of Items	Average Kappa Value	Example topics
A. Identification Information	3	0.81	Gender, marital status
B. Thinking and communication	7	0.46	Hearing, vision, cognition
C. Well-being	8	0.48	Mood, social relationships
D. Daily Activities	22	0.70	ADL, IADL
E. Health Conditions	21	0.65	Falls, substance use, physical symptoms
F. Disease Diagnoses	11	0.50	Miscellaneous diagnoses
G. Nutrition	3	0.32	Weight loss, eating patterns
H. Procedures/Treatments	1	0.31	Influenza vaccination
I. Finances and stressor	2	0.58	Economic trade-offs, stressful events

**Fig. 1:**
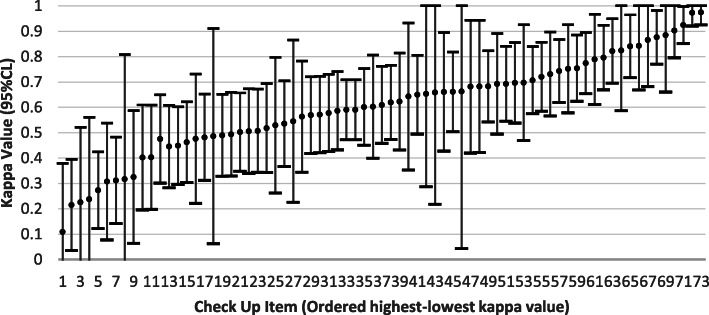
Kappa (95% CL) values for self-reported vs clinician-rated Check-Up items on the interRAI Check-Up instrument

Figure 1 shows the item-specific kappa values ordered from the item with lowest agreement to highest agreement. Of the 74 items considered, 11 had values that fell below 0.40 and 34 had values of 0.65 or greater. Six of the 11 items with low kappa values had highly skewed distributions that could not be further collapsed to ensure more than 5% of cases in one level of a binary variable. The remaining items with low kappas, but non-skewed distributions were: daily decision making, participation in social activities, family being overwhelmed, aphasia, and influenza vaccination. Three of those had kappa values greater than 0.30. The full list of items and associated kappas is included in Additional file 1.

## Discussion

Our results showed that the large majority of Check-Up self-report items completed by the untrained assessors had acceptable to excellent agreement with the ratings of the trained clinician assessor. This indicates that the self-report has a sufficient level of clinical validity to be used for screening in primary care settings in LMICs. That should also be true for higher income nations where the self-report instrument could have value despite a more robust supply of health professionals (for example, in remote or isolated regions, as a pre-surgical screening tool, or as part of health surveys of the general population).

Skewed distributions accounted for some of the variables falling below acceptable kappas. Given that this was a relatively functionally independent population that was able to participate in club activities,^2^ we had less variance than we might see in a population with more physical and cognitive disabilities. This meant that some response levels were sparsely populated and had to be collapsed after the fact. However, it is important to retain the granularity of the Check-Up self-report instruments in order to identify small subset of persons with more severe impairment levels than are typical in the larger target population targeted for screening.

The results of this study are comparable to what has been reported when two clinicians do ratings independently using items from other assessment instruments in the interRAI suite [16, 35].

Our findings provide sufficient evidence to proceed with use of the self-report instrument in South Africa and higher resource nations where literacy levels are higher. However, further testing would be appropriate before using the instrument in countries with different cultures (e.g., India and China). Given that the instrument relies on self-reported items, there is greater risk that cultural factors may bias responses in ways that are less problematic in clinician-administered assessments.

Our findings, as well as our experiences from training additional lay assessors for additional research sites, showed that 8 h of training is sufficient for lay assessors. However, we would recommend periodic follow-up and support based on any issues emerging in the data and to ensure that processes and coding procedures are followed correctly. Depending on the context, electronic support materials (e.g., slides or videos) for assessors to use as a reference source on an ongoing basis would also be useful.

The main limitations of this study include: a modest sample size with limited distributions in some clinical items; inclusion of a single jurisdiction in one country; and lack of follow-up data for examining predictive validity. All of these limitations are being addressed in subsequent research efforts in South Africa and other countries.

## Conclusion

Given that the Check-Up can be administered accurately by non-healthcare professionals, our study bears great promise for the use of multi-domain assessments at scale within low-resource settings using community health workers. The widespread use of the Check-Up tool in these settings can provide valuable data for clinical decision-making where healthcare professionals do not have the capacity to adequately engage with older persons and their complex health needs. Even in high income nations there are some communities with few health resources or geriatric expertise due to low population densities and geographic isolation. In that sense, the Check-Up has potential value for use with disadvantaged populations on a global basis. A scalable assessment model also provides opportunities for the collection of high-level data for understanding the health needs of older persons and healthcare service planning. Further, the self-report instrument can be used outside of clinical settings to determine health needs and functional status of older persons living within communities.

## Supplementary information

**Additional file 1.**

## Data Availability

The datasets used and/or analysed during the current study are available from the corresponding author on reasonable request.

## Abbreviation

LMICs: Low and Middle Income Countries

## Footnotes

1 This number is likely to have increased from 2011 figures given high rates of migration to the area and population ageing – the population of older persons in South Africa increased 56% from 2002 to 2019 to around 9% [32]

2 Twenty-four participants were unable to participate in club activities due to frailty and were assessed in their own homes.
