# Supplementary material for: “Establishing the criterion validity of the interRAI Check-Up Self-Report instrument”
Source: BMC Geriatr. 2020 Jul 29;20:260. doi: 10.1186/s12877-020-01659-9 (PMC7391526; doi:10.1186/s12877-020-01659-9)
Supplement: Supplementary file 1 — Additional file 1. [file 12877_2020_1659_MOESM1_ESM.docx]

*Appendix 1: Full list of items and associated Kappa values*

| **Item Label** | **Kappa** | **Weighted Kappa** |
| --- | --- | --- |
| One or fewer meals a day | 0.1086 | 0.1086 |
| Making self understood | 0.1015 | 0.1452 |
| Family overwhelmed | 0.2147 | 0.2147 |
| Hours exercise | 0.0547 | 0.2177 |
| Aphasia | 0.2317 | 0.3038 |
| Influenza vaccine | 0.3071 | 0.3071 |
| Cancer | 0.3169 | 0.3169 |
| Coronary Heart Disease | 0.249 | 0.3241 |
| Physician visit - 90 day | 0.2255 | 0.3696 |
| Dyspnea | 0.4496 | 0.3868 |
| Daily Decision Making | 0.27 | 0.3939 |
| Decrease in food or fluid | 0.4025 | 0.4025 |
| Diarrhea | 0.3939 | 0.4095 |
| Short-term Memory | 0.2495 | 0.4108 |
| Bowel continence | 0.4792 | 0.418 |
| Social activities | 0.1798 | 0.4204 |
| Major life stressors | 0.4453 | 0.4453 |
| Weight loss | 0.4621 | 0.4621 |
| Change in social activities | 0.415 | 0.4749 |
| Flare-up | 0.4815 | 0.4815 |
| Drove car | 0.4861 | 0.4861 |
| CHF | 0.4344 | 0.4867 |
| ER visit | 0.501 | 0.501 |
| Phone use - Capacity | 0.3814 | 0.5116 |
| Vomiting | 0.5608 | 0.5192 |
| Pain intensity | 0.3285 | 0.5207 |
| Living Arrangement | 0.4536 | 0.5371 |
| Self report: Little interest | 0.4029 | 0.5448 |
| Fatigue | 0.2978 | 0.561 |
| Ability to see in adequate light | 0.4071 | 0.5625 |
| Locomotion - performance | 0.3438 | 0.5692 |
| Ability to understand others | 0.436 | 0.5699 |
| Change in Decision Making | 0.3803 | 0.5901 |
| Change in ADL status (0-2,8) | 0.3803 | 0.5901 |
| Pain frequency | 0.446 | 0.5909 |
| Self report: Anxious | 0.4157 | 0.5958 |
| Overnight hospital stay | 0.598 | 0.598 |
| Walking - performance | 0.3517 | 0.599 |
| COPD | 0.5771 | 0.5998 |
| Unsteady gait | 0.451 | 0.6 |
| Unstable conditions | 0.451 | 0.6 |
| Wound care | 0.4907 | 0.6244 |
| Finance - Capacity | 0.4271 | 0.6384 |
| Chest pain | 0.5304 | 0.6418 |
| Eating - performance | 0.6585 | 0.6585 |
| Loneliness | 0.429 | 0.6604 |
| Other dementia | 0.6626 | 0.6626 |
| Self report: Sad | 0.3953 | 0.6791 |
| Days went out | 0.54 | 0.6886 |
| Hearing | 0.6122 | 0.693 |
| Stairs - Capacity | 0.3271 | 0.6931 |
| Close friend in community | 0.6966 | 0.6966 |
| Locomotion outside of home | 0.3781 | 0.699 |
| Trade-offs | 0.7203 | 0.7203 |
| Managing medications - Capacity | 0.4155 | 0.7295 |
| Self-rated health | 0.5637 | 0.7313 |
| Primary mode of locomotion | 0.6511 | 0.7377 |
| Bladder continence | 0.4463 | 0.7411 |
| Toilet use - performance | 0.7413 | 0.7413 |
| Dressing lower body - performance | 0.5328 | 0.7495 |
| Difficulty falling asleep | 0.5711 | 0.7548 |
| Constipation | 0.5927 | 0.7714 |
| Shopping - Capacity | 0.5566 | 0.7805 |
| Falls 31-90 days ago | 0.6053 | 0.7807 |
| Transfer toilet - performance | 0.5053 | 0.792 |
| Dizziness | 0.5701 | 0.7941 |
| Hours of informal care | 0.7956 | 0.7956 |
| Peripheral edema | 0.5748 | 0.7957 |
| Meal preparation - Capacity | 0.711 | 0.7986 |
| Stroke | 0.7494 | 0.7996 |
| Alcohol | 0.7394 | 0.8323 |
| Bathing - performance | 0.4906 | 0.8393 |
| Housework - Capacity | 0.6798 | 0.8596 |
| Falls last 30 days | 0.8089 | 0.8616 |
| Smokes tobacco | 0.8653 | 0.8653 |
| Transportation - Capacity | 0.7242 | 0.906 |
| Bed mobility - performance | 0.7071 | 0.9064 |
| Hygiene - performance | 0.5457 | 0.9107 |
| Diabetes Mellitus | 0.8551 | 0.9152 |
| Marital Status | 0.9442 | 0.9723 |
| Gender | 1 | 1 |
| Residential Status-Usual | 1 | 1 |
| Alzheimer's | 1 | 1 |
